# Effect of Essential Oils on Growth Inhibition, Biofilm Formation and Membrane Integrity of *Escherichia coli* and *Staphylococcus aureus*

**DOI:** 10.3390/antibiotics10121474

**Published:** 2021-11-30

**Authors:** Andrés Martínez, Marcela Manrique-Moreno, Maria C. Klaiss-Luna, Elena Stashenko, German Zafra, Claudia Ortiz

**Affiliations:** 1 Grupo de Investigación en Bioquímica y Microbiología (GIBIM), Escuela de Microbiología, Facultad de Salud, Universidad Industrial de Santander, Bucaramanga 680002, Colombia; jerson2198175@correo.uis.edu.co (A.M.); geralzaf@uis.edu.co (G.Z.); 2 Grupo de Bioquímica Estructural de Macromoléculas, Instituto de Química, Facultad de Ciencias y Exactas y Naturales, Universidad de Antioquia, Medellín 050010, Colombia; marcela.manrique@udea.edu.co (M.M.-M.); maria.klaiss@udea.edu.co (M.C.K.-L.); 3 Research Center form Biomolecules (CIBIMOL), Escuela de Química, Facultad de Ciencias, Universidad Industrial de Santander, Bucaramanga 680002, Colombia; elenastashenko@gmail.com

**Keywords:** biofilm, antimicrobial activity, essential oil, lipid phase transitions

## Abstract

Biofilm as a cellular conformation confers survival properties to microbial populations and favors microbial resistance. Here, we investigated the antimicrobial, antibiofilm, antimotility, antihemolytic activity, and the interaction with synthetic membranes of 15 essential oils (EOs) on *E. coli* ATCC 25922 and *S. aureus* ATCC 29213. Antimicrobial activity of EOs was determined through microdilution method; development of the biofilm was assessed using the crystal violet assay and SEM microscopy. Results indicate that *Lippia origanoides* thymol–carvacrol II chemotype (LTC II) and *Thymus vulgaris* (TV) exhibited a significant antibacterial activity, with MIC values of 0.45 and 0.75 mg/mL, respectively. The percentage of biofilm formation inhibition was greater than 70% at subinhibitory concentrations (MIC_50_) for LTC II EO. The results demonstrate that these two oils had significantly reduced the hemolytic effect of *S. aureus* by 54% and 32%, respectively, and the mobility capacity by swimming in *E. coli* with percentages of decrease of 55% and 47%, respectively. The results show that LTC II and TV EOs can interact with the hydrophobic core of lipid bilayers and alter the physicochemical properties of membranes. The findings suggest that LTC II and TV oils may potentially be used to aid in the treatment of *S. aureus* and *E. coli* infections.

## 1. Introduction

Antimicrobial resistance (AR) reduces the chances of effective treatment against infectious diseases, representing a worldwide public health problem. One important factor contributing to bacterial resistance is the ability of bacteria to form biofilms [1]. Biofilm is defined as a population of microorganisms attached to a surface, enveloped and organized in an exopolysaccharide matrix that protects it [2,3,4,5]. The biofilm provides a means for sensitive or resistant pathogenic microorganisms to protect themselves against conventional antibiotics, contributing to the chronicity and persistence of infections [6,7,8].

As previously reported [8,9,10,11,12,13], the main mechanisms of antibiotic resistance of a compact biofilm include the formation of a diffusion barrier composed of exopolysaccharides (EPS), reduction in growth velocity, horizontal transfer of resistance genes, physiological latency of persister cells, changes in cell morphology, alteration of pH by generation of microenvironments, and communication systems such as quorum sensing (QS) [8,9,10,11,12,13]. The ability of bacteria to communicate and modulate positive interactions ultimately benefits biofilm formation and promotes host colonization, formation of biofilms, defense against competitors, and adaptation to changing environments [14]. It is in this way that many bacteria form the biofilm. The mechanism of QS depends on bacterial density and secretion of diffusible molecules known as autoinducers, among others [15]. QS plays an important role in inducing gene expression to control different cellular behaviors such as bioluminescence, secretion of virulence factors, biofilm development, and resistance against antimicrobial agents [16,17]. In this sense, it is important to find antimicrobial compounds able to inhibit these metabolic pathways.

*Escherichia coli* and *Staphylococcus aureus* are among the most resistant biofilm-forming pathogenic bacteria. These bacteria occur ubiquitously in water, soil, air, or skin, and can be opportunistic [18]; they constitute one of the most common causes of hospital- and community-acquired infections [19] and have a wide repertoire of virulence factors, among which secreted toxins play a prominent role [20,21]. For example, the pathogenicity of *S. aureus* depends on the production of several virulence factors; in fact, *S. aureus* can express up to 24 cell wall-anchored proteins, which promote adhesion to extracellular matrices, invasion of non-phagocytic cells, biofilm formation, and interference with neutralization of the innate immune system [22,23]. On the other hand, *E. coli* is a causative agent of urinary tract infections (UTI) and tends to form microcolonies in the mucosal membrane of the urinary bladder. These biofilms favor the resistance of the microorganism to the host immune response, increase virulence, and lead to the emergence of resistance to antibacterial drugs by surrounding them in the extracellular biochemical matrix. Furthermore, an interspecies ability to transfer resistance genes through biofilm formation has been demonstrated in *E. coli* [24,25].

Therefore, the search for new therapeutic compounds that interfere with QS mechanisms and biofilm formation has become an interesting focus of study to combat bacterial resistance [19]. Among naturally occurring compounds, essential oils (EOs) from aromatic plants have emerged as an alternative for the treatment of microbial infections [26,27]. EOs are mixtures of low-molecular-weight volatile compounds (e.g., terpenoids and phenols) biosynthesized by plants [28,29]. These products have been of great interest during the last decades for exhibiting broad biological properties, such as antibacterial, antiQS, and antibiofilm effects [30,31,32,33,34,35,36,37].

In this study, the antibacterial, antibiofilm, and antihemolytic effects of 15 EOs distilled from Colombian aromatic plants on planktonic and sessile cells of pathogenic *E. coli* and *S. aureus* strains were evaluated. Synthetic membrane-interaction studies were further conducted using the most active EOs to investigate alterations in cell membrane fluidity on *E. coli* and *S. aureus* representative membrane systems. With the aim of investigating the alteration of cell membrane fluidity, synthetic membrane-interaction studies were performed with the two more active EOs on *E. coli* and *S. aureus* representative membrane systems.

## 2. Results

### 2.1. EO Chemical Composition

The EOs studied in this work were obtained by hydrodistillation, as described elsewhere [38], from fresh plant material cultivated in experimental plots at the Pilot Agricultural Complex (CENIVAM-UIS, Bucaramanga, Colombia). The EO chemical composition was determined using gas chromatography (GC) coupled to flame ionization (FID) and mass selective (MS) detection systems. Two capillary columns of different polarities (polar and nonpolar) were employed to determine linear retention indices (LRI) and to quantify EO components (GC/FID), according to methodology described in previous works [39]. Mass spectra were obtained by GC/MS using electron ionization (EI, 70 eV). Peak matching (coincidence > 90%) of experimental spectra with those from MS databases (Wiley-2008 and NIST-2017) and the study of fragmentation patterns together with the LRIs were the basis for compound identification, the confirmation of which was carried out by using standard terpene compounds found as major components in the EOs studied （Figures S1 and S2）. The major compounds identified in the most active EOs were monoterpenoids thymol, carvacrol, limonene, *p*-cymene, carvone, 1,8-cineole, and geraniol; in a smaller amount, *trans*-caryophyllene, estragole, and benzyl benzoate were found. The results of the EO chemical composition were previously published elsewhere [34,35].

EO Chemical composition can be highly variable, even when they come from the same species. Some studies have shown that the EO chemical composition depends on different factors, including growing conditions, geographical area, age, and plant genetics [40,41]. For example, in the research work [42] on the *Lippia alba* EO chemotype carvone, the relative amount of carvone was 52.8%, and limonene reached 16.98%; while in our study, relative amounts of 31.3% for carvone and 29% for limonene were determined in the EO of *L. alba* chemotype carvone cultivated in Colombia.

### 2.2. EO Antibacterial Activity

**Figure 1:**
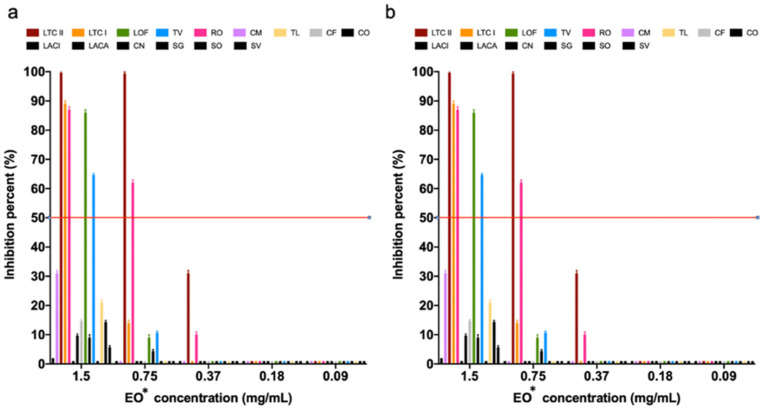
Inhibition effect of EOs on the growth of (**a**) *S. aureus* ATCC 29213 and (**b**) *E. coli* ATCC 25922 for MIC_50_ and MBC determination. Inhibition percentages are presented as the mean ± SD of the absorbance measured at 595 nm with respect to the bacterial growth control. * LTC II (*L. origanoides* thymol–carvacrol II chemotype), TV (*Thymus vulgaris*), CN (*Cymbopogon nardus*), LACI (*Lippia alba citral*), SO (*Salvia officinalis*), LTC I (*L. origanoides* thymol–carvacrol I chemotype), CF (*C. flexuosus*), LACA (*L. alba carvona*), LOF (*L. origanoides felandreno*), CM (*C. martini*), RO (*Rosmarinus officinales*), TL (*Tagetes lucida*), SV (*Satureja viminea*), CO (*Cananga odorata*), SG (*Swinglea glutinosa*).

The effect of different concentrations of each of the 15 EOs on the growth of *S. aureus* ATCC 29213 and of *E. coli* ATCC 25922 appears in Figure 1. *Lippia origanoides* (Verbenaceae family) thymol–carvacrol (LTC II chemotype) was the EO that showed the highest growth inhibitory activity on both tested strains, with a minimum inhibitory concentration (MIC_50_) of 0.45 mg/mL and minimum bactericidal concentration (MBC) of 0.75 mg/mL, respectively ( Figure S3 and Table S3). Other EOs such as *Lippia origanoides*, thymol–carvacrol I chemotype, *Thymus vulgaris*, and *Rosmarinus offiicinalis* (both from Labiatae family) showed a simultaneous but less marked inhibitory effect on both strains. On the other hand, the EO acted more selectively on *E. coli*, whereas *Swinglea glutinosa* EO had a higher inhibitory effect on *S. aureus*.

### 2.3. EO Antibiofilm Activity

**Figure 2:**
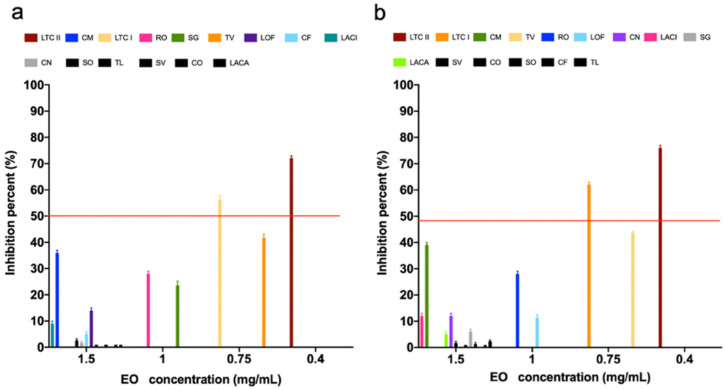
Inhibitory effect of EOs on biofilm formation of (**a**) *S. aureus* ATCC 29213 and (**b**) *E. coli* ATCC 25922. Data are presented as the mean ± SD of absorbance measured at 595 nm relative to the bacterial growth control.

**Figure 3:**
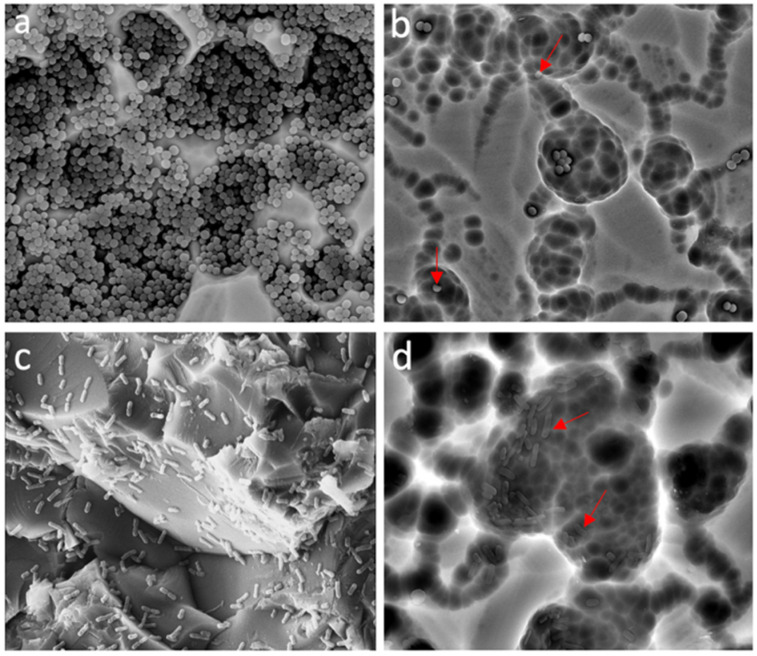
SEM micrographs showing the effect of the LTC II EO on the morphology and structure of the biofilm after 24 h of culture: (**a**) Untreated *S. aureus* ATCC 29213; (**b**) *S. aureus* biofilm treated with LTC II EO; (**c**) Untreated *E. coli* ATCC 25922; and (**d**) *E. coli* ATCC 25922 treated with LTC II EO. Micrographs are presented at 8000× magnification. Red arrows indicate cell membrane shrinkage, deformed cells, and seriously damaged cells.

As presented in Figure 2, most of the tested EOs showed antibiofilm activity on both *S. aureus* ATCC 29213 and *E. coli* ATCC 25922 strains, even though some of these EOs did not show antimicrobial activity. LTC II EO had the highest biofilm inhibitory effect on both tested strains, producing a biofilm formation inhibition of 71% and 76% on *S. aureus* ATCC 29213 and *E. coli* ATCC, respectively (Table S4). On the other hand, the antibiofilm effect of LTC II EO on the morphology and structure of the *S. aureus* ATCC 29213 and *E. coli* ATCC biofilms was evaluated by analyzing treated and untreated samples using scanning electron microscopy (SEM). Figure 3 shows the micrographs of the biofilm of both strains before and after their treatment with the EOs. The results obtained were directly related to the previously quantified antibiofilm activity evaluation (Figure 2 and Figure S4).

### 2.4. EO Effect on the Hemolytic Activity of S. aureus ATCC 29213

**Figure 4:**
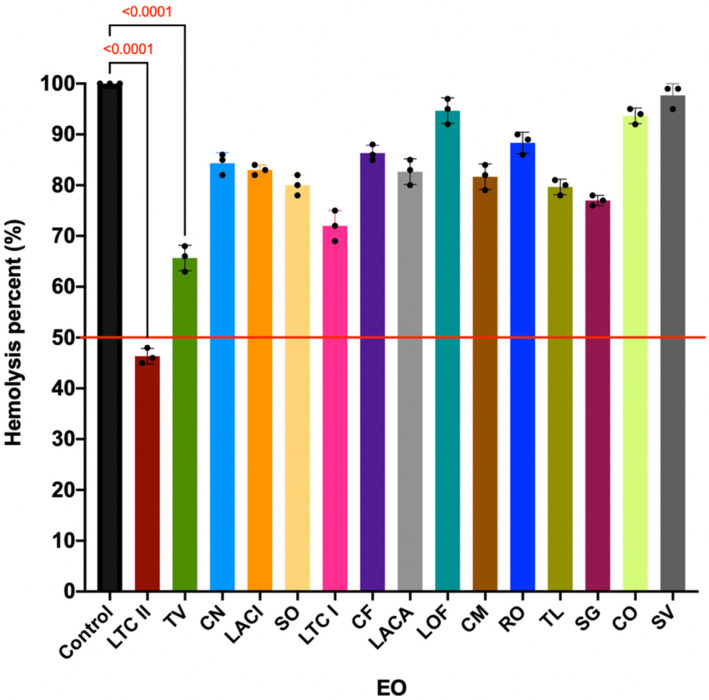
Effect of EOs at subinhibitory concentrations on the hemolytic activity of *S. aureus* ATCC 25922 after 16 h culture in LB medium at 37 °C. Inhibition percentage was calculated considering the hemolytic activity of the control culture (without EO treatment). All experiments were performed in triplicate and analyzed by ANOVA (*p* > 0.05). The ordinary statistical one-way ANOVA analysis between LTC II and TV vs. control showed a *p* value ≤ 0.0001).

The EOs of LTC II and TV significantly reduced the hemolytic effect of *S. aureus* ATCC 29213 by 54% and 35%, respectively (Figure 4, Figure S5 and Table S5) (*p* ≤ 0.0001). This result is interesting because different types of hemolysins produced by this bacterium have been shown to increase the ability of the infection to establish and remain in humans. In addition, it has been shown that the production of these toxins is associated with a possible activation of QS, prior to biofilm formation [43,44,45].

### 2.5. EO Effect on Swimming Motility of E. coli ATCC 25922

**Figure 5:**
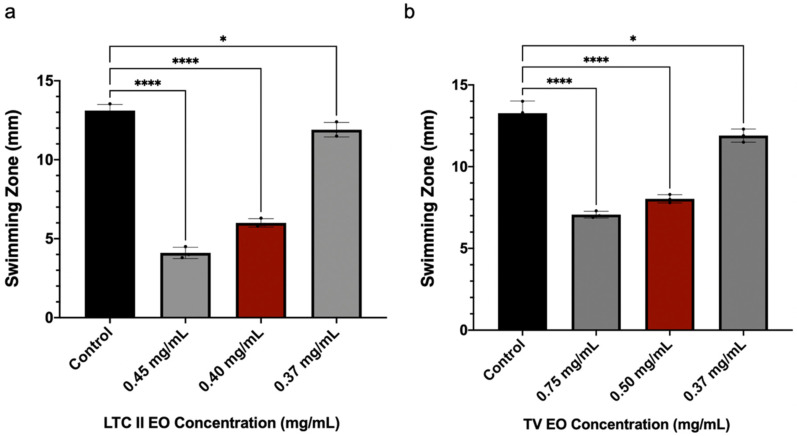
Effect of subinhibitory concentrations of the EOs of (**a**) LTC II and (**b**) TV on the swimming motility of *E. coli* ATCC 25922. Cultures were incubated for 24 h using semi-solid LB medium at 37 °C. The percentage inhibition was calculated by measuring the growth halo in mm, considering the swimming produced by the control culture (untreated). All experiments were performed in triplicate and analyzed by one-way ANOVA. **** *p* ≤ 0.0001, * *p* ≤ 0.0158.

Motility allows *E. coli* to migrate to a new area, facilitating biofilm expansion. Therefore, the swimming motility capacity of *E. coli* ATCC 25922 was evaluated in the presence or absence of the EOs, which showed the highest inhibitory activities (LTC II and TV). The presence of swimming was measured as the diameter (in mm) of the zone of expansion [46]. As shown in Figure 5, subinhibitory concentrations of LTC II and TV inhibited *E. coli* swimming motility by 55% and 47%, respectively, for both oils. These results are interesting, considering that according to other studies [47,48], a very close relationship was established between swimming motility and biofilm formation in *E. coli*.

### 2.6. Phase Transition Experiments of Representative E. coli and S. aureus Model Membranes

The EO antimicrobial activity has been associated with the lipophilic character of monoterpenoids present in them. The wavenumber of the peak position of the νsCH_2_ band as a function of the temperature is a recognized parameter sensitive to lipid order and packing of membrane [49]. The peak positions of the methylene stretching modes νsCH_2_ in each phase of the lipids have different values; in the gel phase νsCH_2_ lies around 2850 cm^−1^ and in the liquid crystalline phase around 2853 cm^−1^. The Tm has a characteristic value for each phospholipid depending on the length of the acyl chains [50] and on the structure of the head groups [51]. However, to accurately represent the *E. coli* and *S. aureus* membrane behavior, we prepared liposomes built of the most representative lipids of each bacterial system. In the case of *E. coli*, a mixture of phosphatidylethanolamine and phosphatidylglycerol (POPE/POPG, 70:30) was selected for being most representative of the lipids [52].

**Figure 6:**
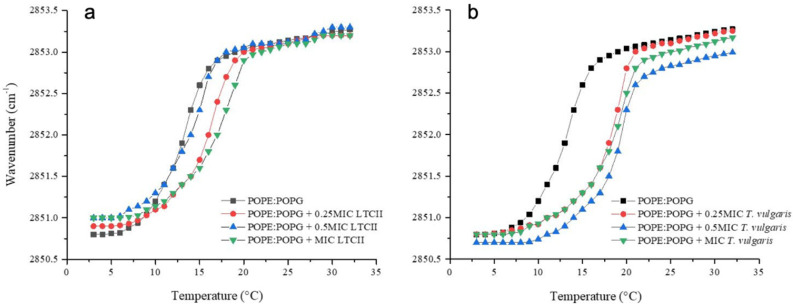
Peak positions of the symmetric stretching vibration bands of the methylene groups as a function of temperature. *E. coli* representative model membrane (POPE/POPG, 70:30) in the presence of different concentrations of (**a**) LTC II and (**b**) TV EOs.

Figure 6 shows the temperature dependence of the wavenumber values of the peak positions of the POPE/POPG acyl chains for the *E. coli* lipid system and for the mixtures at different concentrations of LTC II and TV EOs. Lipid systems in the absence of essential oils presented a Tm of 13 °C. The interaction of both EOs with representative lipid systems of *E. coli* resulted in an increase in the Tm as well as in a more packed system (less fluid). The strongest alteration of the *E. coli* lipid system was produced by TV EO, where Tm was shifted by as much as 5 °C up to 18 °C at the highest concentration evaluated. At the same concentration, LTC II EO induced a shift of ca. 3.5 °C. The *S. aureus* representative system was built with phosphatidylglycerol and cardiolipin (DMPG/CL, 80:20) for being the most abundant lipids in membrane [53].

**Figure 7:**
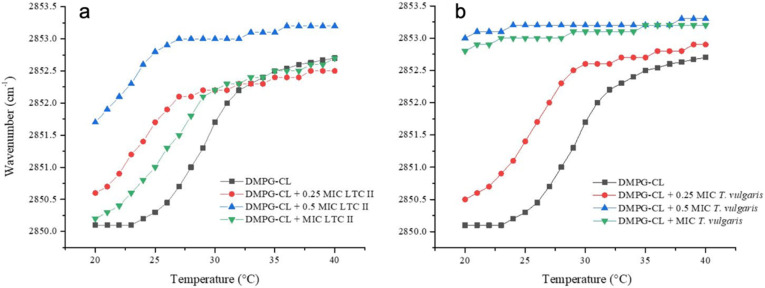
Peak positions of the symmetric stretching vibration bands of the methylene groups as a function of temperature. *S. aureus* representative model membrane (DMPG/CL, 80:20) in the presence of different concentrations of (**a**) LTC II and (**b**) TV EOs.

Figure 7 shows the temperature dependence of the wavenumber values of the peak positions for the DMPG/CL acyl chains for the *S. aureus* lipid system and for the mixtures at different concentrations of LTC II and TV. The lipid systems in the absence of EOs presented a Tm of 29 °C. The interaction of both EOs with representative lipid systems resulted in a decrease in the Tm as well as in the fluidization of the membrane. The strongest alteration was produced again by TV EO, where Tm was reduced by as much as 4 °C at the lowest concentration evaluated, and it was not possible to determine Tm at the other two concentrations. A slight fluidization of the acyl chains was evident from the increase in the wavenumbers of the νsCH_2_ band at fixed temperature, which might give evidence for a direct interaction of the TV EO with the acyl chains of phospholipid system that alters the lipid packing of liposomes. In the case of LTC II EO, the effect was less destabilizing, but the effect also had important consequences for the membrane packing.

## 3. Discussion

The EOs tested in this study showed an inhibitory effect against *E. coli* ATCC 25922 and *S. aureus* ATCC 29213 bacterial growth, as presented in Figure 1 and  Table S2. Different studies [26,27,28] have shown that the antimicrobial effect is directly related to the major compounds in the EOs. However, the minor components could have an antagonistic, synergistic, or neutral effect depending on the studied microorganism [54]. The mechanisms of EO action depend on its chemical composition and the synergistic or antagonistic interactions between its compounds [51]. For instance, thymol and carvacrol have similar antimicrobial effects but have different mechanisms of action against Gram-positive and Gram-negative bacteria [55]. In this study, it was found that LTC II and TV EOs presented the highest values of antimicrobial and antibiofilm activities on *S. aureus* ATCC 29213 and *E. coli* ATCC 25922, with MIC values in the range from 0.4 to 1 mg/mL. An antibacterial effect of the *Rosmarinus oficcinalis* and *Swinglea glutinosa* EOs on *E. coli* and *S. aureus* was also evidenced, probably due to the presence of α-pinene and 1,8-cineol, which are associated with membrane effects Table S1 [56,57].

According to the chemical characterization performed in previous studies [34,35], these EOs presented high content of phenolic and monoterpene compounds, such as thymol and carvacrol (Table 1). In addition, cytotoxicity studies performed by our research group [34] showed that LTC II and TV EOs were more selective towards bacterial strains than towards Vero cells, in which case the selectivity indices (SI) were negative.

The biological activities associated with the use of thymol are mainly antioxidant, analgesic, anticonceptive, anti-inflammatory, antifungal, antibacterial, antibiofilm, and anticancer [58,59,60]. One of the possible mechanisms of action that has been proposed for thymol is its ability to disintegrate the outer membrane of Gram-negative bacteria. This releases the lipo-polysaccharide and increases the permeability of the cytoplasmic membrane [61,62]; in this sense, Ferreira et al. tested the effect of thymol on lipid monolayers and analyzed the interaction between thymol and the lipid dipalmitoylphosphatidylcholine (DPPC). The results showed that thymol decreases the surface elasticity and changes the morphology of DPPC, thus demonstrating that the compound embeds between membrane lipids [63]. In the present study, an effect of EO on Gram-positive and Gram-negative membranes was demonstrated.

Regarding the mechanism of action of carvacrol, it has been suggested that it acts as a monovalent cation membrane transporter, exchanging its proton from the hydroxyl group with another ion, such as K^+^; the undissociated (protonated) carvacrol diffuses across the cytoplasmic membrane into the cytoplasm where it dissociates, releasing its proton. This is based on the K^+^ outflow and H^+^ influx observed in Bacillus cereus exposed to carvacrol [64,65]. The second most abundant component in TV EO was ρ-cymene (20%); its biological properties are broad, including antioxidant, anti-inflammatory, anticancer, anxiolytic, and antimicrobial effects [66]. This last property has been evaluated by different authors [26,34], and it has been determined that ρ-cymene is not the main compound that confers antimicrobial activity to EOs; however, it enhances the activity of other antimicrobial compounds, exerting a synergistic effect.

Even though all 15 EOs produced different degrees of biofilm inhibition on *E. coli* ATCC 25922 and *S. aureus* ATCC 29213, the LTC II and TV EOs notably produced the highest inhibitory effects on sessile but not on planktonic cells. SEM images also showed a disruption of the three-dimensional structure of mature biofilms after treatment with the LTC II EO, and it was evident that cell density was higher in untreated than in EO-treated biofilms. In addition, SEM photomicrographs showed the effect of LTC II EO interaction on cell membrane integrity, such that cell lysis, deformation, and swelling were observed (Figure 3), suggesting membrane permeabilization. Previous studies [67] have demonstrated the effect of EOs with anti-biofilm activity on Gram-positive and Gram-negative bacteria. Among the biofilm inhibitory effects associated with this type of natural product, the following stand out: quorum sensing inhibition, in turn decreasing the expression of virulence factors and bacterial adherence to different surfaces [68], adhesins and exopolysaccharides production [69]; collapse of proton motive force due to ATPase inhibitory activity; decrease in energy in the form of ATP; and blocking of substance flow [70].

In addition, it has been shown that EOs could inhibit cellular communication pathways, such as QS, hindering biofilm formation [33]. In the present study, we demonstrated that the LTC II and TV EOs reduced the hemolytic effect of *S. aureus* ATCC 25922 (Figure 4). Other studies [27,33] have shown that EOs produce the alteration of the agr QS system of *S. aureus*. This effect would be directly related to the production of PIA and PNAG (exopolysaccharide), adhesins and extracellular proteins, associated with biofilm development [44,71]. On the other hand, *S. aureus* produces a toxin called α-hemolysin that causes hemolysis; the production of this toxin is determined by the regulation of the P3 operon (RNAIII-hla), which is regulated by the QS communication system [43,44,45]. Considering that the activation of the QS system in *S. aureus* by the P2 and P3 operon pathway induces the expression of alpha hemolysin, the decrease in the quantification of alpha hemolysin in the presence of EOs would be considered an indicator of QS inhibition.

On the other hand, the effect of EOs on the motility of *E. coli* ATCC 25922 was demonstrated as presented in Figure 5. These results are related to the affectation of pathways related to flagellar development in the early stages of biofilm formation [72]. Other authors [7,14,73] have demonstrated a relationship between the antibiofilm effect and QS pathways. For example, commensal *E. coli* has several QS pathways, including an indole-based system, which is produced by TnaA from tryptophan, and another system based on autoinducer 2 (AI-2). This autoinducer influences both biofilm formation and motility (swimming motility and chemotaxis). The fact that the *E. coli* AI-2 signal secreted by cells attracts other *E. coli* cells and leads to increased biofilm formation indicates that *E. coli* cells actively seek out other *E. coli* cells to form communities [74].

Recent studies have shown that the mechanisms of action of phenolic monoterpene compounds in EOs are mainly associated with their activity on cell viability, and their interaction with transcriptional regulators of QS communication, biofilm formation, and virulence genes has been proposed [75,76]. In addition, it has been evidenced that the activity of the major components of an EO could be altered by its minor components, either in a synergistic, antagonistic, or neutral way, depending on the relative amount of these minor components [77,78]. Therefore, the effect of an EO could vary between microorganisms and even between different strains of the same microorganism [79,80]. Regarding LTC II and TV EOs, although the individual biological activity of minority components such as γ-terpinene, ρ-cymene, and trans-β-caryophyllene has not yet been reported, it has been observed that together with phenolic monoterpenes they could have synergistic activity, thus dramatically increasing the overall EOs biological activity [81].

To characterize the EOs’ capability of interacting with phospholipidic membranes, two synthetic lipid systems representative of Gram-positive *S. aureus* and Gram-negative *E. coli* were used. The study of the thermotropic phase transition of these systems is a very useful biophysical technique for following the effect of EO components on lipid membrane organization. The results show that the LTC II and TV EOs were able to interact with the hydrophobic core of lipid bilayers and alter the physicochemical properties of membranes. In the case of the Gram-negative lipid system, both EOs showed an increase in the phase transition temperature related to the intercalation of the substances among the acyl chain lipids. On the other hand, in the Gram-positive model, the EO effect was related to the ability to affect the fluidity of the liposomes. At a concentration of 0.5 MIC and MIC of EOs, the effect was so strong that the phase transition of liposomes was lost. These results are in accordance with the results obtained by Ozkan et al., [82] who demonstrated that the carvacrol and thymol hydrophobicity affected the permeability of cell membranes.

Some of the components of the EOs have been tested individually to understand their role in the antimicrobial activity [83,84,85]. However, in the LTC II and TV EOs, the presence of several compounds may have a synergic effect that contributes to the interaction and destabilization of the hydrophobic core of lipid bilayers and therefore with physicochemical properties of membranes. The results obtained with EOs studied are very similar depending on the membrane system, which could be related to the net surface charge of the bacterial membrane. For the *S. aureus* membrane, a more negatively charged surface is characteristic of a Gram-positive bacteria in comparison with the more zwitter-ionic surface of *E. coli* system.

## 4. Materials and Methods

Plants used in this study were harvested from the experimental plots located in the Agroindustrial Pilot Complex of CENIVAM (National Center for Research on Agro-Industrialization of Tropical Medicinal Aromatic Plants) at Universidad Industrial de Santander (Bucaramanga, Colombia). The taxonomic characterization of the plants was carried out in the Institute of Natural Sciences of the Universidad Nacional of Colombia (Bogotá, Colombia). *Staphylococcus aureus* ATCC 29213 and *Escherichia coli* ATCC 25922 strains were commercially purchased by the Grupo de Investigación en Bioquímica y Microbiología (GIBIM), Universidad Industrial de Santander.

Essential oils were extracted by microwave-assisted hydrodistillation (MWHD) in Clevenger-type distillation equipment adapted to a heating system in a Samsung MS-1242zk domestic microwave (Seoul, Korea; oven with an output power of 1600 W and 2.4 GHz radiation frequency). The plants (200 g) suspended in water (300 mL) were placed in a 2 L balloon, which was connected to a Clevenger type glass equipment with a Dean–Stark distillation reservoir. The plant sample was heated by microwave irradiation for 45 min (3 × 15 min, consecutive cycles). The essential oil obtained was dried with anhydrous sodium sulfate, weighed, and stored in an amber bottle at 4 °C until further analysis. All extractions were made in triplicate [39].

### 4.1. Determination of the EO Antimicrobial Activity

Antimicrobial activity of fifteen EOs was carried out as previously described with some modifications [34]. Briefly, in a 96-well microplate, the MIC of essential oils was determined using the broth microdilution method for bacteria. EOs were dissolved in dimethyl sulfoxide (DMSO). Serial dilutions of the EOs were prepared ranging from 1.5 up to 0.09 mg/mL to a final volume of 100 µL per well. All experiments were conducted with a maximum of 1% (*v*/*v*) DMSO in solution. An amount of 100 µL of bacterial suspension was added to each well to obtain a final inoculum concentration of 5.2 × 10^7^ CFU mL and a working volume of 200 µL. Luria Bertani (LB) and Tryptic Soy Broth (TSB) culture media for *E. coli* ATCC 25922 and *S. aureus* ATCC 29213, respectively, were used as growth control. Ofloxacin and vancomycin were used as a positive control. In vitro cultures were incubated at 37 °C with constant agitation during 24 h, and the optical density was monitored at 595 nm in a Bio-Rad iMark microplate absorbance reader version 1.02.01 (Hercules, CA, USA). All experiments were performed in triplicate.

### 4.2. EO Antibiofilm Activity

The EOs were assessed for their potential to inhibit biofilm formation of a biofilm produced by strains *E. coli* ATCC 25922 and *S. aureus* ATCC 29213. Individual wells of sterile polystyrene round bottomed microtiter plates were used, as described previously with some modifications [34]. Cultures were grown overnight in 3 mL TSB with 2% glucose, diluted in growth medium to 4 × 10^6^ CFU/mL for *S. aureus* ATCC 29213, whereas for *E. coli* ATCC 25922, LB culture medium was used in the same proportions. An amount of 100 µL of the respective culture medium was transferred into the plate in the presence of 100 µL subinhibitory concentrations (subMIC) of the EOs. One hundred microliters of culture medium were used as control. After incubation for 24 h at 37 °C, the biofilms were washed three times with sterile phosphate buffer saline (PBS pH 7.2) to remove free-floating planktonic bacteria. Biofilms formed by adherent sessile organisms in plate were stained with crystal violet (0.4% *w*/*v*). All experiments were performed in triplicate and the percentage inhibition was calculated as follows:

Inhibition percentage = (OD negative control − OD treated sample/OD negative control) × 100%

### 4.3. Analysis of Biofilm by Scanning Electron Microscopy (SEM)

Observations of the possible morphological changes in both bacteria were carried out by SEM, following the protocol described by Singh et al., with some modifications [86]. SEM was used to investigate the structural modifications of biofilms after treatment with EOs. Biofilm formation of *E. coli* ATCC 25922 and *S. aureus* ATCC 29213 was carried out on glass coupons (1 cm × 2 cm). The selected coupons were rinsed three times with phosphate-buffered saline (PBS; pH 7.2). Sample preparation for SEM was performed as follows: soaking of the sample with 2.5% glutaraldehyde for 2 h at room temperature. Coupons were washed using different solutions of isopropyl alcohol: 5, 15, 25, 50, 75, and 100% for 5 min each, rinsing at room temperature.

### 4.4. Anti-Hemolytic Activity Assay in S. aureus

An antihemolytic activity assay was performed according to a previously described methodology [87], with some modifications. A culture of *S. aureus* ATCC 29213 was prepared in LB broth, incubated overnight at 37 °C, and orbitally shaken at 200 rpm. The culture was then diluted 100-fold in fresh LB broth, and 100 µL was seeded in 96-well flat-bottom microplates containing 100 µL EO at subinhibitory concentrations. The microplate was incubated for 16 h at 37 °C and kept under orbital shaking at 200 rpm. Negative controls were prepared by mixing 100 µL of culture with 100 µL of 0.1% peptone broth (without EO). Subsequently, the microplate was centrifuged at 4400 rpm for 2 min at 4 °C. For the hemolysis assay, a red blood cell suspension was prepared by centrifuging rabbit blood at 2000 rpm for 2 min. The precipitate was recovered, and three washes were performed with phosphate-buffered saline (PBS), and subsequently, a 1% *v*/*v* red blood cell suspension was prepared in PBS. To assess hemolysis, 100 µL of the supernatant of the *S. aureus* culture seeded in the microplate was mixed with 100 µL of the rabbit suspension and incubated at 37 °C for one hour. Subsequently, the samples were centrifuged at 4400 rpm for 10 min, and the supernatant was recovered to finally measure absorbance at a wavelength of 543 nm. Controls of the EOs mixed with the red blood cell suspension were prepared to confirm that the EOs did not cause hemolysis.

### 4.5. Bacterial Swimming Motility Assay in E. coli

The swimming motility assay was performed as previously described, with some modifications [72,88]. For this assay, a culture of *E. coli* ATCC 25922 was prepared in LB broth and incubated overnight at 37 °C under orbital shaking at 200 rpm. The culture was then diluted 100-fold in fresh LB broth and 100 µL was seeded into 96-well flat-bottom microplates containing 100 µL of EO at subinhibitory concentrations. The microplate was incubated for two hours at 37 °C and kept under orbital shaking at 200 rpm. Negative controls were prepared by mixing 100 µL of culture with 100 µL of 0.1% pepton broth (without AE). Subsequently, 2 µL samples were taken from the microplate wells to deep seed Petri dishes with semisolid agar prepared with LB liquid medium and 0.25% agar. They were then kept in incubation at 37 °C for 24 h. At the end of incubation, the diameters of the swimming zone in mm were measured and compared with those of the control culture, which had not been in contact with the EO. Motility assays were performed in triplicate.

### 4.6. Synthetic Lipid Systems of Bacterial Membranes

Based on the membrane lipid composition of *E. coli* and *S. aureus*, major lipid components were selected to obtain the synthetic lipid systems used to phase transition analysis by infrared spectroscopy. According to the literature [89], the *E. coli* membrane is mainly composed of phosphatidylethanolamine (70%) and phosphatidylglycerol (30%) and, in the case of *S. aureus* [53], the main lipids are phosphatidylglycerol (80%) and cardiolipin (20%). (The synthetic lipids used in the experiments were palmitoyl-oleoylphosphatidylethanolamine (POPE, Lot. 160-181PE-139, Avanti Polar Lipids Alabaster, Alabaster, AL, USA), Palmitoyloleoylphosphatidylglycerol (POPG, Lot. 160-181PG-135, Avanti Polar Lipids Alabaster, AL, USA), dimyristoylphosphatidylglycerol (DMPG, Lot. 140PG-167, Avanti Polar Lipids Alabaster, AL, USA), and cardiolipin (CL, Lot. 750332P-200MG-A-030, Avanti Polar Lipids Alabaster, AL, USA). These lipids were selected because their main transition temperatures are in the AquaSpec temperature range.

### 4.7. Infrared Spectroscopy Experiments

Lipid suspensions were prepared to attain a concentration equivalent to 20 mM. The corresponding amount of lipid was dissolved in chloroform, and then the solvent was removed under N_2_ stream to obtain a lipid film. The lipid films were hydrated for 30 min in buffer (20 mM HEPES, pH 7.4 for *E. coli* or Hepes 20 mM, NaCl 500 mM, and EDTA 1 mM pH 7.4 for *S. aureus*) in a sonicator at 37 °C. For the phase transition measurements, the background acquisition was performed using a buffer in a programmed temperature ramp from 5 to 50 °C at a heating rate of 1 °C/min with an equilibration of 120 s between each measurement. One hundred twenty interferograms were accumulated for each temperature. For the lipid mixture, 20 µL of the liposome suspension was injected into an AquaSpec cell coupled to a Tensor II spectrometer (Bruker, Karlsruhe, Germany) with an MCT detector. The experiments in the presence of the EOs at different concentrations were performed, adding the EOs to the chloroform before forming the film to allow their incorporation into the liposomes. The following steps were carried out as described above. The L_β_–L_α_ phase transition was monitored using the symmetric peak vibration of the methylene groups of the acyl chains in the window of 2970 to 2820 cm^−1^ and plotted as a function of temperature.

### 4.8. Data Analysis

All experiments were performed in triplicate and one-way analysis of variance (ANOVA) was performed to analyze the results among treatments. The significance level in each assay was <0.05%. The assumption of normality and data variances were previously tested using the Shapiro–Wilk and Levene test, respectively.

## 5. Conclusions

In the present study, it was demonstrated that the *L. origanoides* (thymol/carvacrol chemotype, LTC II) and *Thymus vulgaris* essential oils have both antibacterial and antibiofilm activity on *E. coli* ATCC 25922 and *S. aureus* ATCC 29213, suggesting that they also have anti-QS activity. The LTC II was the EO producing the highest effects on biofilm development and key bacterial communication mechanisms such as QS, which were confirmed with the decrease in the hemolytic effect of *S. aureus* and the decrease in swim motility in *E. coli*. Biophysical experiments using synthetic membranes made it possible to demonstrate that the EO liposomal models can interact with the lipid environment of the membranes, can modify the transition temperature, and may serve as EOs transport vehicles.

## Figures and Tables

**Table 1:** Main compounds present in the EOs that showed the highest antibacterial and antibiofilm activities, expressed in relative quantity [32,33].

Code	Plant Species	Chemotype	Majority Constituents
LTC II	*Lippia origanoides*	thymol–carvacrol II	γ-terpinene (5.2%), *p*-cymene (1.1%), thymol (32.7%), carvacrol (18.8%), and *trans*-β-caryophyllene (6.4%)
TV	*Thymus vulgaris*	-	γ-terpinene (9.5%), *p*-cymene (20%), linalool (4.7%), *trans*-β-caryophyllene (9.5%), and thymol (23%).

## Data Availability

Data are contained within the article.

## Supplementary Materials

The following are available online at https://www.mdpi.com/article/10.3390/antibiotics10121474/s1, Figure S1: Total ion current (TIC, chromatogram) obtained by GC/MS (electron ionization, 70 eV) of the Lippia origanoides (Verbenceae family) hydrodistilled-essential oil, Figure S2: Total ion cur-rent (TIC, chromatogram) obtained by GC/MS (electron ionization, 70 eV) of the Thymus vulgaris (Labiatae family) hydrodistilled-essential oil, Figure S3: Antibacterial activity of the LTC II EO against the growth rate of E. coli ATCC 25922 (a) and S. aureus ATCC 29213 (b), Figure S4: Colony Forming Unit (CFU/mL) of planktonic cells exposed to sub-MIC concentrations of LTC II in anti-biofilm experiments of a) E. coli ATCC 25922 and b) S. aureus ATCC 29213, Figure S5: Colony Forming Units presented (CFU/mL) presented in log10, of the S. aureus ATCC 29213 planktonic cells treated and not treated with CTL II at subinhibitory concentrations of 0.40 mg / mL in the anti-hemolytic activity, Table S1: Major metabolites present in the EOs assessed. Relative amount of each metabolite is reported in percentage (%), Table S2: Minimum Inhibitory Concentration able to inhibiting 50% of bacterial growth (MIC50) and Minimum Bactericidal Concentration (MBC) in mg/mL determined for evaluated Eos, Table S3: Colony-forming units (CFU/mL), log reduction and inhibition percentage of bacterial growth of different concentrations of LTC II, Ta-ble S4: Colony-forming units (CFU/mL), log reduction and inhibition percentage of biofilm for-mation of different concentrations of LTC II, Table S5: Antihemolytic effect of the 17 AEs against S. aureus ATCC.
