# Supplementary material for: Effect of Essential Oils on Growth Inhibition, Biofilm Formation and Membrane Integrity of Escherichia coli and Staphylococcus aureus
Source: Antibiotics (Basel). 2021 Nov 30;10(12):1474. doi: 10.3390/antibiotics10121474 (PMC8698458; doi:10.3390/antibiotics10121474)
Supplement: Supplementary file 1 [file antibiotics-10-01474-s001.zip › antibiotics-1432878-supplementary.pdf]

Effect of Essential Oils on Growth inhibition, Biofilm Formation and Membrane Integrity of *Escherichia coli* and *Staphylococcus aureus*

Andrés Martínez, Marcela Manrique-Moreno, Maria C. Klaiss-Luna, Elena Stashenko, German Zafra[4], and Claudia Ortiz.

Supplemental material

Table S1. Major metabolites present in the EOs assessed. Relative amount of each metabolite is reported in percentage (%).

Abbrev.	Plant Species	Chemotype	Principal metabolites
LACA	*Lippia alba*	Carvona	Limonene (29%), β-bourbonone (2.4%), germacrene D (12.2%), carvone (31.3%), and piperitenone (1.5%)
LACI	*Lippia alba*	Citral	Limonene (3.9%), *trans*-β-caryophyllene (11.8%), neral (15.4%), geranial (18.9%), and geraniol (6.1%)
CN	*Cymbopogon nardus*		Citronellal (11.6%), 2,6-dimethyl-2,6-octadiene (6.1%), β-citronellol (16.9%), and geraniol (17.8%)
CM	*Cymbopogon martini*		*trans*-β-Ocimene (1.9%), linalool (3.2%), geranyl acetate (1.3%), and geraniol (38.7%)
CF	*Cymbopogon flexuosos*		Neral (24.5%), geranial (33%), geraniol (7.9%), and geranyl acetate (0.5%)
LOTC I	*Lippia origanoides*	timol-carvacrol I	p-Cymene (3.7%), thymyl methyl ether (4.6%), *trans*-β-caryophyllene (7.9%), thymol (22.1%), carvacrol (10.7%), and thymyl acetate (3.9%)
LOTC II	*Lippia origanoides*	timol-carvacrol II	γ-Terpinene (5.2%), p-cymene (1.1%), thymol (32,7%), carvacrol (18.8%), and *trans*-β-caryophyllene (6.4%)
LOF	*Lippia origanoides*	Felandreno	α-Phellandrene (5.7%), 1,8-cineole (11.6%), p-cymene (5.7%), *trans*-β-caryophyllene (10.4%), and α-humulene (6.2%).
RO	*Rosmarinus offiicinalis*		α-Pinene (12.7%), camphene (7.7%), 1,8-cineole (17.5%), camphor (14.8%), and *trans*-β-caryophyllene (7.8%).
SO	*Salvia officinalis*		1,8-Cineole (5.3%), *trans*-thujone (20.4%), *cis*-thujone (5.8%), camphor (8.5%), and α-humulene (9.8%).
SG	*Swinglea glutinosa*		α-Pinene (2.6%), *trans*-β-caryophyllene (36.6%), germacrene D (15.4%),

			germacrene B (10.8%), and *trans*-nerolidol (24.0%).
TL	*Tagetes lucida*		Estragole (79.9%) and β-myrcene (0.9%).
TV	*Thymus vulgaris*		γ-Terpinene (9.5%), p-cymene (20%), linalool (4.7%), *trans*-β-caryophyllene (9.5%), and thymol (23%).
SV	*Satureja viminea*		1-Isopropenyl-4-methyl-1-ciclohexane (24.4%), *trans*-β-caryophyllene (11.8%), pulegone (11.1%), and *cis*-pulegol (7.1%).
CO	*Cananga odorata*		Linalool (11.7%), methyl benzoate (3.7%), benzyl acetate (10.3%), (Z)-cinnamyl acetate (5.4%), and benzyl benzoate (20.8%).

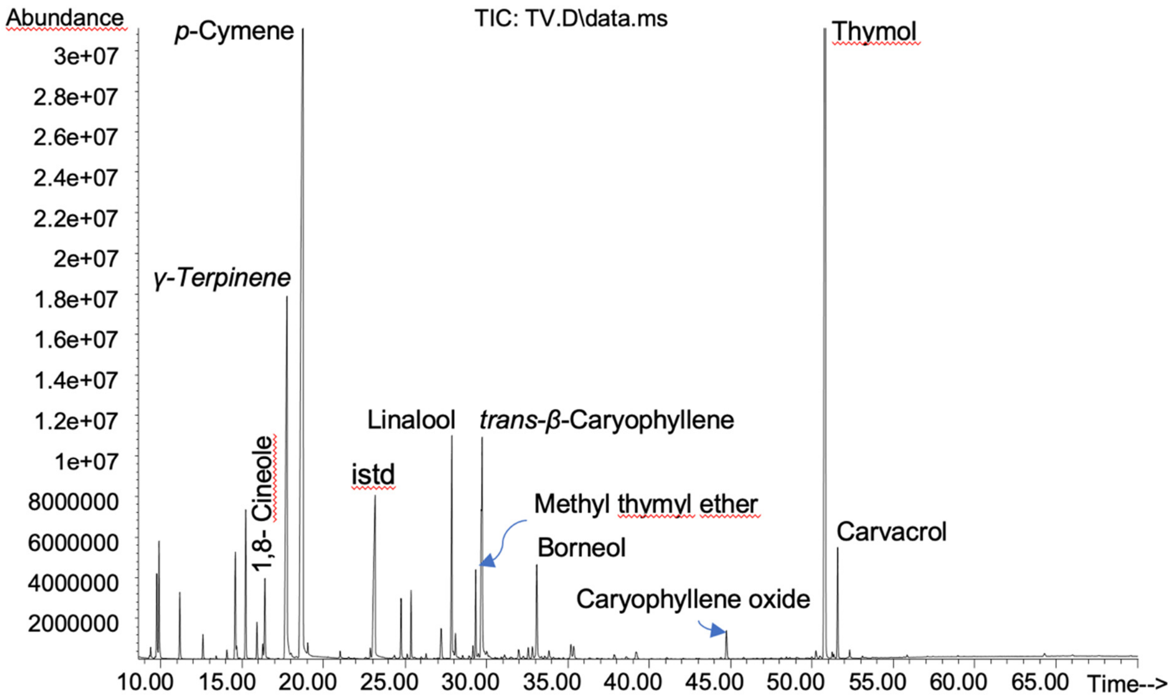

Figure S1. Figure 1S. Total ion current (TIC, chromatogram) obtained by GC/MS (electron ionization, 70 eV) of the *Lippia origanoides* (Verbenceae family) hydrodistilled-essential oil. Polar DB-WAX (60 m) column. Split 1:30. Istd – internal standard (*n*-tetradecane).

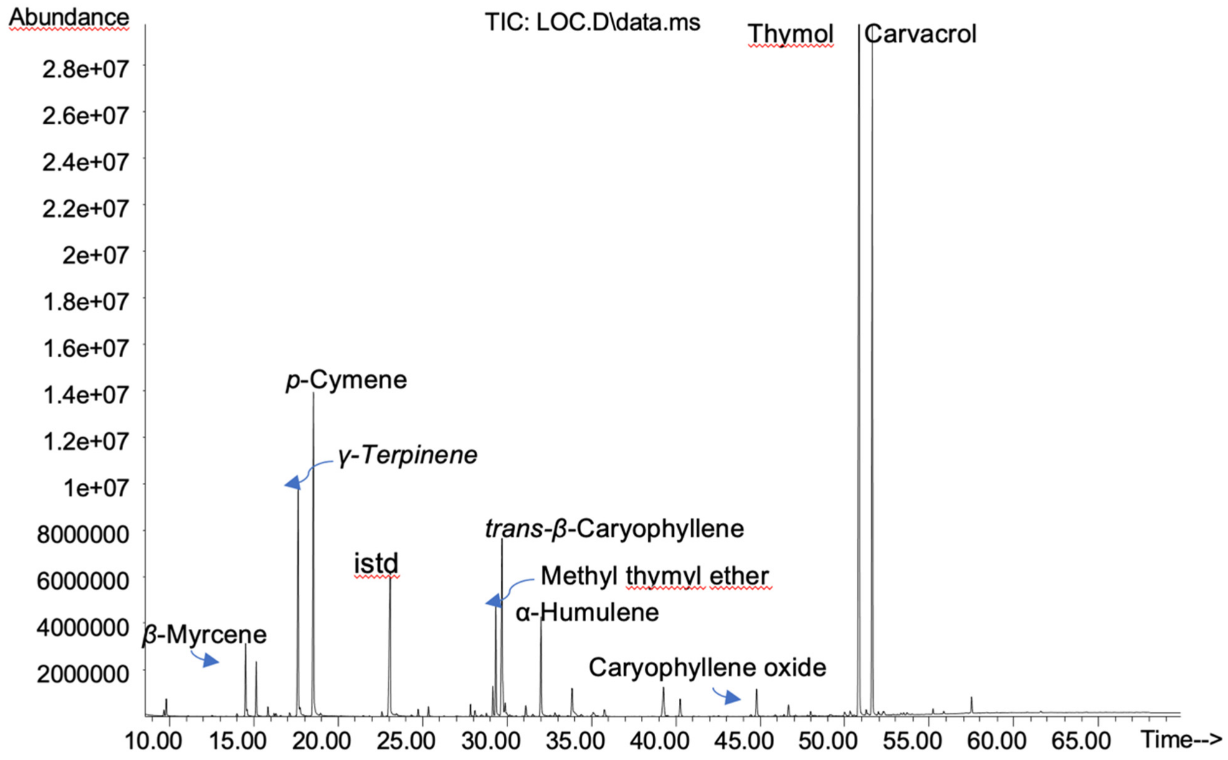

Figure S2. Total ion current (TIC, chromatogram) obtained by GC/MS (electron ionization, 70 eV) of the *Thymus vulgaris* (Labiatae family) hydrodistilled-essential oil. Polar DB-WAX (60 m) column. Split 1:30. Istd – internal standard (*n*-tetradecane).

Table S2. Minimum Inhibitory Concentration able to inhibiting 50% of bacterial growth (MIC_{50}) and Minimum Bactericidal Concentration (MBC) in mg/mL determined for evaluated EOs. Experiments were performed in triplicate.

	***E. coli* ATCC 25922**		***S. aureus* ATCC 29213**	
**Essential oils**	**MBC (mg/mL)**	**MIC_{50} (mg/mL)**	**MBC (mg/mL)**	**MIC_{50} (mg/mL)**
Lippia alba citral	> 1.5	>1.5	> 1.5	> 1.5
Cymbopogon martini	> 1.5	> 1.5	> 1.5	> 1.5
Lippia origanoides LTC II	0.75	0.45	0.75	0.45
Lippia origanoides LTC I	1	0.75	1	0.75
Rosmarinus offiicinalis	1.5	1	1.5	1
Lippia alba carvona	> 1.5	> 1.5	> 1.5	> 1.5
Cymbopogon nordus	> 1.5	> 1.5	> 1.5	> 1.5
Cymbopogon flexuosus	> 1.5	> 1.5	> 1.5	> 1.5

Lippia origanoides felandreno	1.5	1	> 1.5	> 1.5
Salvia officinalis	>1.5	>1.5	>1.5	>1.5
Swinglea glutinosa	> 1.5	> 1.5	1.5	1
Tagetes lucida	> 1.5	> 1.5	> 1.5	> 1.5
Thymus vulgaris	1	0.75	1	0.75
Satureja viminea	> 1.5	> 1.5	> 1.5	> 1.5
Cananga odorata	> 1.5	> 1.5	> 1.5	> 1.5

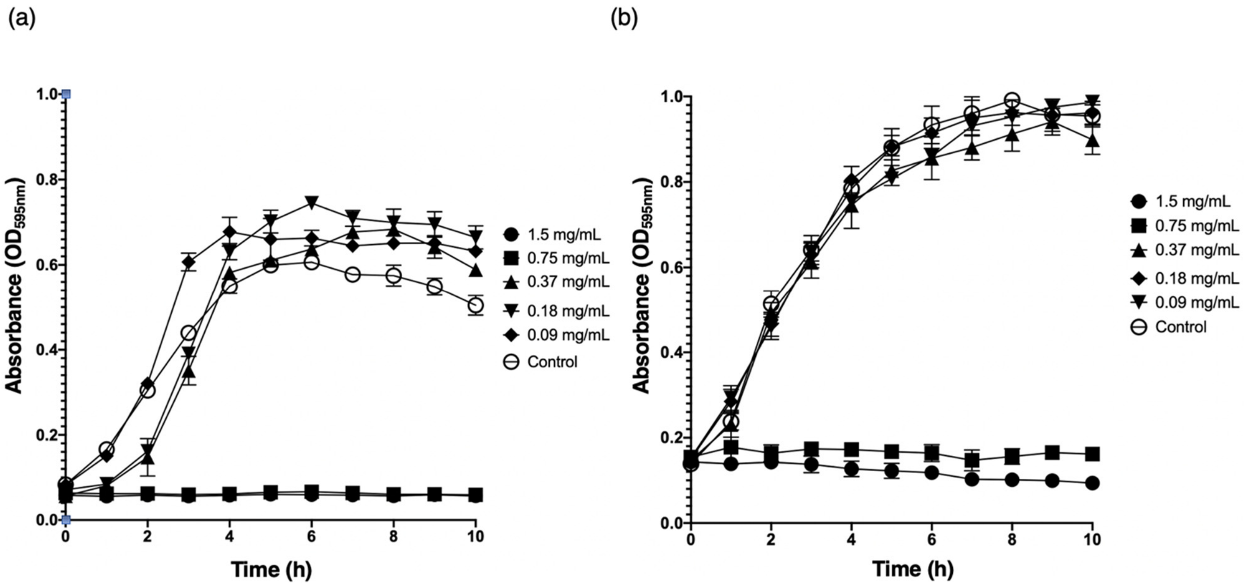

Figure S3. Antibacterial activity of the LTC II EO against the growth rate of *E. coli* ATCC 25922 (a) and *S. aureus* ATCC 29213 (b). Data are presented as the mean ± SD of absorbance measured at 595 nm.

Table S3. Colony-forming units (CFU/mL), log reduction and inhibition percentage of bacterial growth of different concentrations of LTC II

	E. coli ATCC 25922			*S. aureus* ATCC 29213		
LTC II concentration (mg/mL)	Planctonyc CFU/mL	Log reduction	Inhibition percent (%)	CFU/mL	Log reduction	Inhibition percent (%)
0.37	5 x 10^6	0	0	4.1 x 10^8	0	0
0.40	3.9 x 10^6	0	0	3.6 x 10^8	0	0
0.45	1.9 x 10^6	0.82	56	1.8x 10^8	0.75	53
0.50	3.5 x 10^5	2.51	92	2.4 x 10^7	2.79	94

Table S4. Colony-forming units (CFU/mL), log reduction and inhibition percentage of biofilm formation of different concentrations of LTC II

	E. coli ATCC 25922			*S. aureus* ATCC 29213		
LTC II concentration (mg/mL)	Sessile cells (CFU/mL)	Log reduction	Inhibition percent (%)	CFU/mL	Log reduction	Inhibition percent (%)
0.37	4.4×10^6	0.1	24	3.4×10^8	0.2	19
0.40	3.6×10^6	0.3	76	1.2×10^8	1.2	72
0.45	2.5×10^5	2.9	94	2.7×10^7	2.7	95

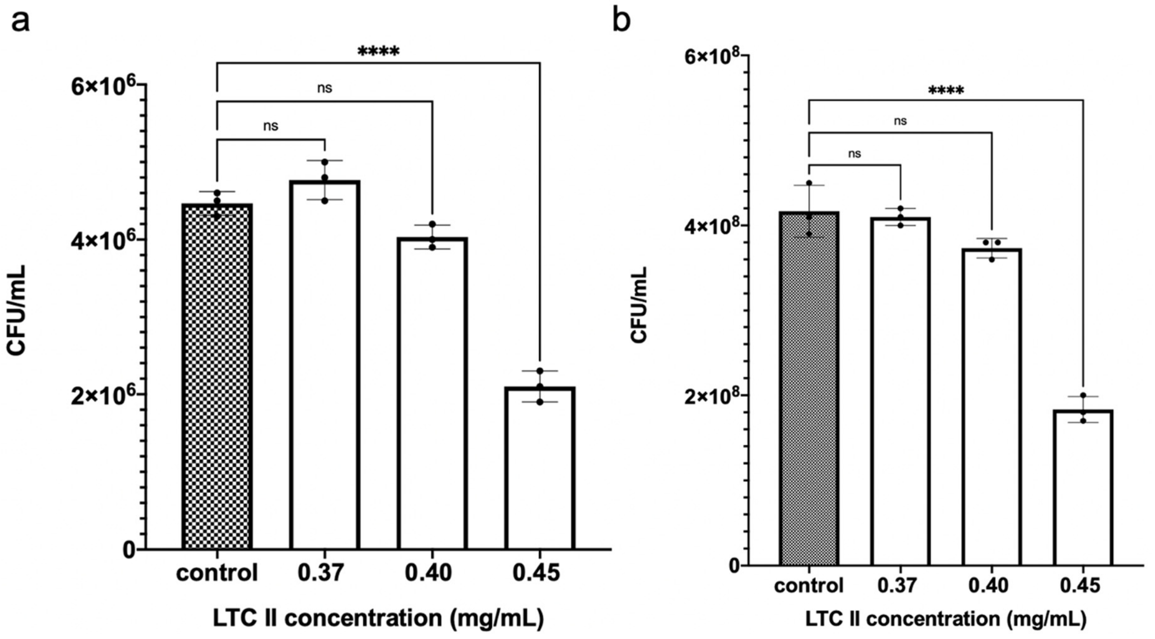

Figure S4. Colony Forming Unit (CFU/mL) of planktonic cells exposed to sub-MIC concentrations of LTC II in antibiofilm experiments of a) *E. coli* ATCC 25922 and b) *S. aureus* ATCC 29213. Each experiment was performed in triplicate and an ANOVA was performed.

Table S5. Antihemolytic effect of the 17 AEs against *S. aureus* ATCC. Each experiment was performed in triplicate and was expressed as a percentage of inhibition. Inhibition percentage was calculated taking into account raw results OD of the control and the treatment.

Essential oil	EO concentration (mg/mL)	Hemolytic percent (%)	Planktonic cells (CFU/mL)
Lippia alba citral	1.5	83	2.0×10^8
Cymbopogon martini	1.5	82	1.8×10^8
Lippia origanoides LTC II	0.4	46	1.7×10^8

Lippia origanoides LTC I	0.75	72	2.0×10^9
Rosmarinus offiicinalis	1	88	2.4×10^8
Lippia alba carvona	1.5	83	2.8×10^8
Cymbopogon nordus	1.5	84	2.1×10^8
Cymbopogon flexuosus	1.5	86	2.4×10^8
Lippia origanoides felandreno	1.5	95	1.9×10^8
Salvia officinalis	1.5	80	2.5×10^8
Swinglea glutinosa	1	77	1.7×10^8
Tagetes lucida	1.5	80	2.0×10^9
Thymus vulgaris	0.75	66	1.8×10^8
Satureja viminea	1.5	98	2.3×10^8
Cananga odorata	1.5	94	2.0×10^9
Control (*S. aureus* ATCC 29213)	-	100	2.2×10^8

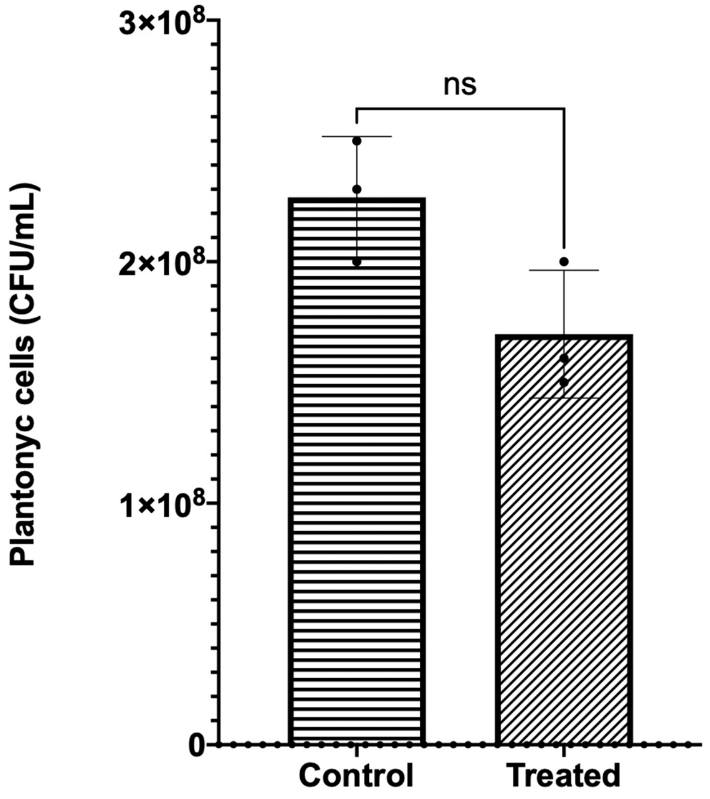

Figure S5. Colony Forming Units presented (CFU/mL) presented in log10, of the *S. aureus* ATCC 29213 planktonic cells treated and not treated with CTL II at subinhibitory concentrations of 0.40

mg / mL in the anti-hemolytic activity. The experiments were performed in triplicate and T-test was performed as statistical analysis.
